# c-Myc affects mRNA translation, cell proliferation and progenitor cell function in the mammary gland

**DOI:** 10.1186/1741-7007-7-63

**Published:** 2009-09-28

**Authors:** Tina Stoelzle, Patrick Schwarb, Andreas Trumpp, Nancy E Hynes

**Affiliations:** 1 Friedrich Miescher Institute for Biomedical Research, Basel, Switzerland; 2 Division of Stem Cells and Cancer, German Cancer Research Center (DKFZ), Heidelberg, Germany; 3 HI-STEM (Heidelberg Institute for Stem Cell Technology and Experimental Medicine), Heidelberg, Germany

## Abstract

**Background:**

The oncoprotein c-Myc has been intensely studied in breast cancer and mouse mammary tumor models, but relatively little is known about the normal physiological role of c-Myc in the mammary gland. Here we investigated functions of c-Myc during mouse mammary gland development using a conditional knockout approach.

**Results:**

Generation of *c-myc*^*fl*/*fl *^mice carrying the mammary gland-specific *WAPiCre *transgene resulted in c-Myc loss in alveolar epithelial cells starting in mid-pregnancy. Three major phenotypes were observed in glands of mutant mice. First, c-Myc-deficient alveolar cells had a slower proliferative response at the start of pregnancy, causing a delay but not a block of alveolar development. Second, while milk composition was comparable between wild type and mutant animals, milk production was reduced in mutant glands, leading to slower pup weight-gain. Electron microscopy and polysome fractionation revealed a general decrease in translational efficiency. Furthermore, analysis of mRNA distribution along the polysome gradient demonstrated that this effect was specific for mRNAs whose protein products are involved in milk synthesis. Moreover, quantitative reverse transcription-polymerase chain reaction analysis revealed decreased levels of ribosomal RNAs and ribosomal protein-encoding mRNAs in mutant glands. Third, using the mammary transplantation technique to functionally identify alveolar progenitor cells, we observed that the mutant epithelium has a reduced ability to repopulate the gland when transplanted into NOD/SCID recipients.

**Conclusion:**

We have demonstrated that c-Myc plays multiple roles in the mouse mammary gland during pregnancy and lactation. c-Myc loss delayed, but did not block proliferation and differentiation in pregnancy. During lactation, lower levels of ribosomal RNAs and proteins were present and translation was generally decreased in mutant glands. Finally, the transplantation studies suggest a role for c-Myc in progenitor cell proliferation and/or survival.

See related minireview by Evan et al:

## Background

The oncoprotein c-Myc is a basic helix-loop-helix transcription factor implicated in multiple cellular processes, including proliferation, differentiation, metabolism, and apoptosis (reviewed in Eilers and Eisenman [1]). c-Myc regulates RNA polymerase II (Pol II) driven transcription of a large set of targets [2-4] and has been reported to have effects on global chromatin modification [5]. Furthermore, c-Myc stimulates RNA Pol I [6,7] and Pol III [8,9] mediated transcription, thus linking it to ribosome biogenesis and translation. In addition, c-Myc has been implicated in mitochondrial biogenesis [10] and global miRNA expression [11]. Recently, non-transcriptional effects of c-Myc on DNA replication [12] and translation progression [13] have also been described.

Deregulated levels of c-Myc, resulting from amplification, translocation, transcriptional, translational as well as other mechanisms have been observed in numerous human tumors (reviewed in Vita and Henriksson [14]). In breast cancer, c-Myc overexpression occurs in >50% of primary tumors [15] and has been reported to correlate with poor prognosis [16]. The use of transgenic mouse models has helped to analyze c-Myc-induced mammary tumorigenesis [17-19], but little is known about the normal physiological function of c-Myc in the mammary gland.

A number of studies have described different roles for c-Myc in other organs. The full knockout of c-Myc is embryonic lethal [20,21], due to its indispensable function in the placenta and the hematopoietic system [22,23]. Several conditional mouse models expressing Cre recombinase under different promoters have been generated in order to delete c-Myc in skin [24,25], liver [3,26,27], pancreas [28,29], intestines [30,31], and bone marrow [32-34]. Taken together, the results revealed various organ-specific roles for c-Myc in controlling development and regeneration, cell size or number and stem cell differentiation and maintenance. Each report is of interest, not only for deciphering physiological functions of c-Myc, but also when considering c-Myc as a therapeutic target in human cancer.

The mammary gland is a convenient model for developmental studies, as it goes through repeated cycles of proliferation, differentiation and apoptosis during puberty and pregnancy. The gland of a mature virgin female consists of two compartments, a ductal epithelial network and the stroma or mammary fat pad. Upon hormonal stimulation in pregnancy, bursts of proliferation followed by differentiation allow the gland to convert into a milk-synthesizing machine. To study the role of c-Myc in the mammary gland, a conditional approach using the Cre-loxP system was employed. Whey acidic protein *(WAP)iCre *transgenic mice were used to recombine the LoxP-flanked *c-myc *locus in luminal alveolar cells starting at mid-pregnancy and throughout lactation. Following loss of c-Myc in the mammary gland, three main phenotypes were observed. At the start of pregnancy, c-Myc-deficient alveolar cells were impeded in their proliferative response resulting in a delayed ability to differentiate. Moreover, mutant glands displayed lower expression levels of ribosomal RNA and proteins as well as a general decrease in translation. Finally, the mutant mammary epithelium had a reduced ability to grow when transplanted into mammary fat pads. These results suggest that c-Myc has multiple roles in the mammary gland, affecting proliferation, biosynthetic capacity, and progenitor cell proliferation and/or survival.

## Results

### *WAPiCre*-mediated ablation of c-Myc in the mammary gland

**Figure 1:**
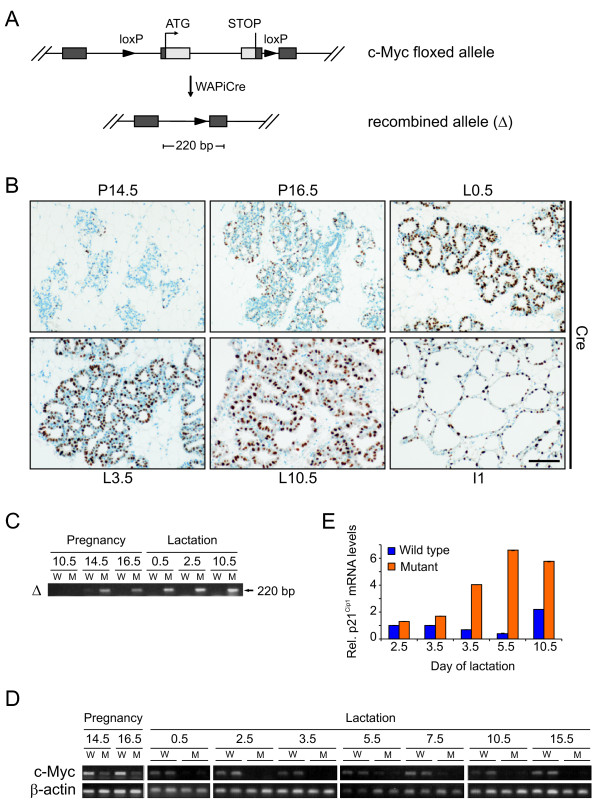
**Targeted disruption of c-Myc in the mammary gland**. **(a) **Schematic diagram of *c-myc *floxed allele and recombined allele after Cre-mediated excision of floxed region. The position of the 220 base pair (bp) polymerase chain reaction (PCR) product for detecting recombined allele is indicated. **(b) **Immunohistochemistry against Cre (brown nuclei) on paraffin sections of mutant mammary glands. Representative staining from different stages of pregnancy (P), lactation (L), and involution (I). Scale bar, 100 μm. **(c) **PCR on genomic DNA from glands taken at the indicated days from wild type (WT) (W) and mutant (M) mice to detect the recombined *c-myc *allele (220 bp) indicated in (a). **(d) **Semi-quantitative reverse transcription-PCR showing *c-myc *and *β-actin *mRNA levels in glands of WT and mutant mice removed at two time points during a first pregnancy and at seven times in lactation. **(e) **Relative expression levels of *p21*^*Cip*1 ^determined by qPCR in WT and mutant glands at four different time points in lactation. Results are the average of duplicate measurements with *β-actin *mRNA levels as reference.

To study the role of c-Myc in mammary gland development, we used a conditional approach, crossing *c-myc*^*fl*/*fl *^mice [21] to *WAPiCre *transgenic mice [35]. The generated offspring will be referred to as wild type (WT, *c-myc*^*fl*/*fl*^;*WAPiCre*^- ^or *c-myc*^*fl*/+^;* WAPiCre*^-^), heterozygous (*c-myc*^*fl*/+^;*WAPiCre*^+^) and mutant (*c-myc*^*fl*/*fl*^;*WAPiCre*^+^) mice. In animals positive for the *WAPiCre *transgene, the complete open reading frame of *c-myc *will be excised upon Cre expression (Figure 1(a)). To assess onset and extent of *WAPiCre *expression, we performed immunohistochemistry (IHC) against Cre recombinase on sections from mutant mammary glands (Figure 1(b)). Cre expression was first detected at day 14.5 of pregnancy in scattered luminal alveolar cells. The number of Cre-expressing cells increased continuously until after parturition, when positive staining for Cre was seen in essentially all luminal cells. To monitor recombination, we performed polymerase chain reaction (PCR) on genomic DNA isolated from mammary glands at different developmental stages. The 220 base pair band, indicating the presence of the recombined allele, was first detected at day 14.5 of pregnancy (Figure 1(c)), consistent with the results from IHC. Starting then, levels of *c-myc *mRNA decreased rapidly in glands of mutant mothers and were essentially undetectable throughout lactation (Figure 1(d)). With the commercially available antibodies, it has not been possible to detect c-Myc in the lactating mammary gland by IHC (data not shown; Klinakis *et al*. [36]). Since the half-life of c-Myc protein and mRNA is short [37], it is likely that mutant glands have little or no c-Myc by the onset of lactation. Finally, mRNA levels of the cell cycle inhibitor *p21*^*Cip*1^, a well-studied target of c-Myc-mediated repression [38,39], were upregulated in c-Myc-deficient glands during lactation (Figure 1(e)), which is in agreement with the functional loss of c-Myc in mutant glands.

### c-Myc mutant mothers display a lactation defect with less efficient milk production

**Figure 2:**
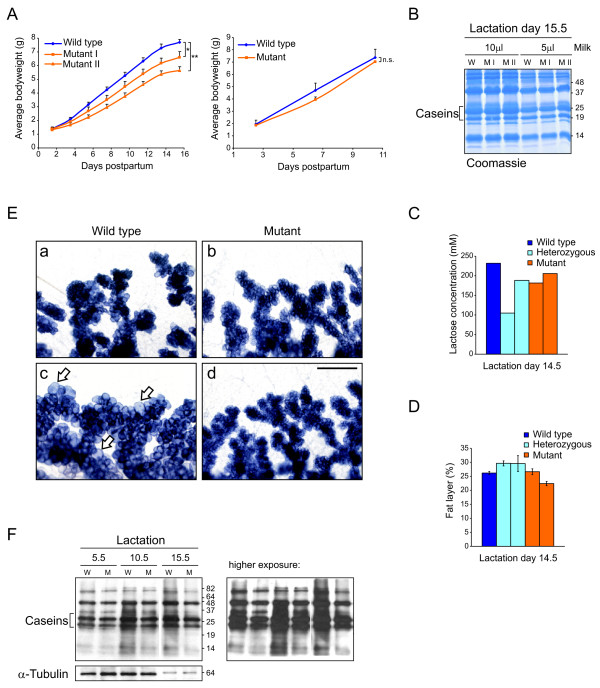
**Ablation of c-Myc in mammary glands results in impaired lactation due to reduced milk volume**. **(a) **Growth analysis of pups nursed by wild type (WT) or mutant mothers. Data are shown as average body weight plus standard deviation. Left panel: analysis of three littermate mothers nursing seven WT pups each. *, *P *= 2.2 × 10^-5^; **, *P *= 1.1 × 10^-9^. Right panel: comparison of a WT mother with six pups to a mutant mother nursing two pups (all pups WT littermates). NS = not significant, *P *= 0.52. **(b) **Milk protein composition in milk obtained from WT (W) or mutant (M) mice at lactation day 15.5. Freshly collected milk was diluted 1:20 in phosphate-buffered saline and 5 or 10 μl loaded on a 15% SDS-gel for Coomassie staining. **(c) **Measurement of lactose concentration in milk collected at lactation day 14.5. In a colorimetric assay, lactose concentration is determined as concentration of free galactose in lactase-treated skim milk. Results from five animals with the indicated genotype are shown as average value of duplicate measurements. **(d) **Analysis of fat content in the same milk samples used in (c), presented as percentage of fat layer length over total length of milk. Results are the mean ± standard deviation of three measurements per animal. **(e) **Whole mounts of WT and mutant mammary glands collected on lactation day 0.5. Mothers were sacrificed directly after removing them from pups (a, b), or after 2 hours without pups to allow filling of glands with milk (c, d). Arrows point to distended alveoli in WT gland. Scale bar, 500 μm. **(f) **Western analysis of milk proteins (loading: 1 μg per lane) and α-tubulin (loading: 9 μg per lane of the identical lysates) of WT and mutant mammary gland lysates at lactation day 5.5, 10.5, and 15.5. Blot was probed with anti-milk serum; two different exposure times are shown.

Monitoring survival and weight of newborn pups is routinely used as a measure of lactation [40]. Thus, we performed a pup weight analysis to examine the efficiency of milk production in WT and mutant females. Growth curves generated from seven foster pups per mother showed that pups nursed by mutant mothers grew significantly slower compared with pups nursed by a WT mother (Figure 2(a), left panel). However, when comparing a mutant mother nursing only two foster pups to a WT mother nursing six pups, there was no significant difference in pup body weight (Figure 2(a), right panel). This suggests that milk quantity, but not quality might be affected in c-Myc-deficient glands.

To test this hypothesis, we first examined milk composition. Milk samples taken from WT and mutant mothers at day 14.5 and 15.5 of lactation were analyzed for protein, lactose and fat content, the three major milk components. On a Coomassie stained gel, milk protein pattern and concentration were identical in equal volumes of milk from WT and mutant mothers (Figure 2(b), caseins are indicated) (see also Marte *et al*. [41]). Furthermore, the concentration of lactose, the major carbohydrate and osmole in milk, as well as the fat content were determined in milk samples from a group of five animals made up of WT, heterozygous (showing no overt phenotype) and mutant mothers (Figure 2(c) and 2(d)). Lactose concentration was determined in a colorimetric assay on skim milk samples, whereas fat content was measured as the ratio of cream layer length over total milk length after centrifugation ('creamatocrit') (Lucas *et al*. [42]). While one heterozygous mother showed a slightly decreased lactose concentration, likely due to natural variation (Figure 2(c)), there were no consistent alterations in either lactose or fat content within the samples.

Next, to compare the approximate amount of milk produced in the lactating glands from WT and mutant mothers, the following experiment was performed. In the first setting, mothers were sacrificed immediately after removing them from their actively suckling pups. In the second setting, mothers were removed from their pups and sacrificed 2 hours later, which allows the glands to fill with milk. When comparing high magnifications of whole mount preparations taken from actively nursing mothers, glands from WT and mutant mice looked nearly identical (Figure 2(e), panels a and b). However, only the WT females showed clear signs of milk-filling, displaying large, distended alvoeli after 2 hours without pups (Figure 2(e), panel c, arrows), while glands of mutant mothers appeared only slightly distended (Figure 2(e), panel d).

Finally, we examined the milk proteins via a Western analysis carried out on protein lysates made from lactating mammary glands of WT and mutant mothers. Equal amounts of protein were loaded and membranes probed with a rabbit anti-milk serum [41], producing a staining pattern of multiple milk proteins (Figure 2(f)). The blot shows that mutant protein lysates contain less milk protein than the corresponding WT lysate at day 5.5, 10.5, and 15.5 of lactation. The level of α-tubulin, used as a loading control, was the same in each paired WT and mutant sample. Taken together, these results clearly suggest that the reduced nursing ability in c-Myc mutant mothers is due to decreased or slower milk production, while milk composition is essentially the same in mutant and WT mothers.

### Alterations in alveolar density and secretory activity

**Figure 3:**
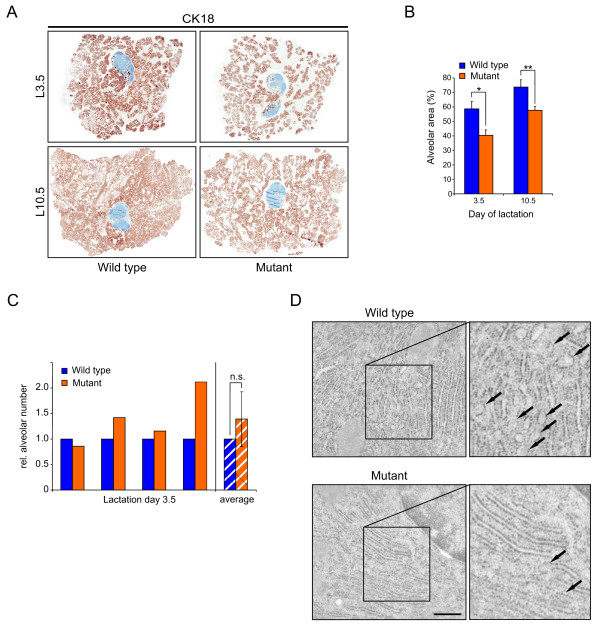
**Alterations in alveolar density and secretory activity**. **(a) **Immunohistochemistry against cytokeratin (CK) 18 on sections of wild type (WT) and mutant glands to visualize epithelium (brown). For optimal comparison, a central part of each gland containing the lymph node was taken. Lymph nodes (blue) and stroma display no CK18 staining. **(b) **Quantification of alveolar area per total organ area (excluding lymph node) from sections processed as shown in (a). Two sections per animal were quantified and the average was used for calculations. Results are the mean ± standard deviation of four and three pairs of animals quantified on lactation day 3.5 and 10.5, respectively. *, *P *= 0.00057; **, *P *= 0.0043. **(c) **Number of alveoli (including mean ± standard deviation) measured in four pairs of animals at lactation day 3.5, which were used for area measurement in (b). Numbers were calculated per analyzed area, and values from WT glands were set to 1. NS = not significant, *P *= 0.25. **(d) **Electron microscopy pictures from day 7.5 lactating WT and mutant glands. Arrows point to regions of dilated, vesicle forming endoplasmic reticulum. Scale bar, 500 nm.

To analyze the lactation defect in more detail, we investigated the morphology of glands from lactating, actively nursing WT and mutant mothers via IHC. Cytokeratin (CK) 18-stained cross-sections were scanned (Figure 3(a)), revealing that mutant glands contained more unstained stromal area than WT glands. To obtain quantitative results, the area covered by alveoli (including epithelium and lumen) was measured and the ratio of alveolar area over total organ area was calculated (Figure 3(b)), revealing that alveolar area in mutant glands was significantly decreased, on average by 30% (lactation day 3.5) and 20% (lactation day 10.5). Interestingly, the number of alveoli per organ area was not altered (Figure 3(c)), suggesting that in mutant glands there is a reduction in the size of alveoli, which could be due to smaller and/or fewer alveolar cells or less milk, even in an active nursing state.

To analyze this in more detail, proliferation and apoptosis were investigated in lactating glands. We did not detect any difference in BrdU incorporation between WT and mutant glands (data not shown), nor were shed cells apparent in the lumens (for example, when looking at high magnifications of Figures 1(b) and 3(a)), suggesting no dramatic alterations in cell number. Thus, we examined the glands via electron microscopy to look directly at the secretory activity of alveolar cells. The endoplasmic reticulum forms highly organized, parallel strands, from which secretory vesicles bud to fuse into the alveolar lumen (Figure 3(d)). When comparing day 7.5 lactating WT and mutant glands, mutant cells are dominated by parallel regions of thin regular endoplasmic reticulum. In contrast, WT cells contain more dilated reticulum and budding vesicles (arrows), indicating high protein synthesis activity. The result was confirmed in two pairs of day 4.5 lactating mice (not shown). The non-dilated endoplasmic reticulum in c-Myc mutant glands suggests a defect in protein synthesis, at the cellular level.

### c-Myc controls biosynthetic activity in the mammary gland

**Figure 4:**
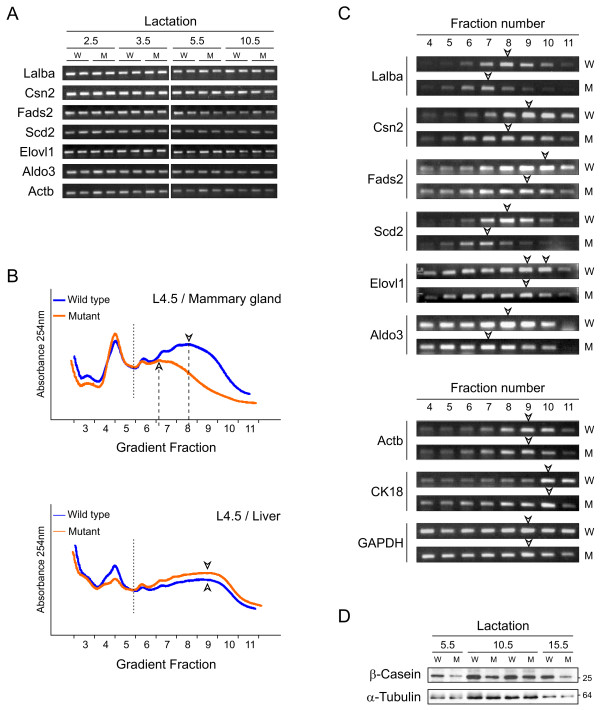
**Altered translation efficiency in mutant mammary glands**. **(a) **Semi-quantitative reverse transcription-polymerase chain reaction (RT-PCR) on *α-lactalbumin (Lalba)*, *β-casein (Csn2)*, *Δ6 fatty acid desaturase 2 (Fads2)*, *stearoyl-CoA desaturase 2 (Scd2), elongation of very long chain fatty acids (Elovl1)*, *aldolase C (Aldo3) *and *β-actin (Actb) *in wild type (WT) (W) and mutant (M) glands taken at different time points during lactation. **(b) **Polysome profiles of day 4.5 lactating mammary glands (upper panel) and corresponding livers (lower panel) of the same animals. Profiles were overlaid according to their 80S peak. Hollow arrowheads mark the peaks of the polysomal fractions. **(c) **Semi-quantitative RT-PCR on mRNA isolated from the indicated gradient fractions from WT and mutant polysomes. mRNA distribution analyzed for genes described in (a) (upper panel) and 'control genes' *β-actin*, *CK18*, and *GAPDH *(lower panel). Hollow arrowheads indicate the peak of each mRNA distribution along the gradient. **(d) **Western analysis of β-casein (loading: 0.25 μg per lane) and α-tubulin (loading: 50 μg per lane of the identical lysates) on WT and mutant mammary gland lysates obtained at three time points in lactation.

The previous results suggest that c-Myc loss in alveolar cells might cause a general defect in milk production, including milk components and the enzymes involved in their synthesis. This was analyzed in more detail, first by examining mRNA levels of milk proteins and enzymes that are strongly upregulated in lactating mammary glands [43,44]. Transcripts encoding: α-lactalbumin (Lalba) and β-casein (Csn2), both milk proteins, the former also the rate-limiting co-factor for lactose synthesis [45], as well as Δ6 fatty acid desaturase 2 (Fads2), stearoyl-CoA desaturase 2 (Scd2), elongation of very long chain fatty acids (Elovl1), and aldolase C (Aldo3), enzymes involved in lipid synthesis [44], were measured by semi-quantitative reverse transcription (RT)-PCR. All transcripts were expressed at comparable levels in WT and mutant glands analyzed between lactation day 2.5 and 10.5 (Figure 4(a)), including Fads2, Scd2 and Elovl1 that are described Myc targets in other systems (, Zeller *et al*. [46]). This suggests that regulation of milk production by c-Myc might occur by a non-transcriptional mechanism.

Next we investigated mRNA translation in WT and mutant glands by performing polysome fractionation on mammary gland lysates obtained at lactation day 4.5. This technique allows the separation of mRNAs along a sucrose gradient depending on their ribosomal load. When overlaying profiles from WT and mutant glands according to their monosome peaks, a change in the average size of polysomes was evident in c-Myc deficient glands, with the peak being shifted to smaller polysomes (Figure 4(b), upper panel). Results from one pair of WT and mutant animals are shown; three additional pairs of animals were examined, yielding similar results (data not shown). As a control, we performed polysome fractionations on livers obtained from the females used for generating the mammary gland profiles. WT and mutant mice retain c-Myc in the liver since *WAPiCre *is not expressed there. The polysome distribution from livers of WT and mutant females was nearly identical (Figure 4(b), lower panel), showing that the altered polysome distribution is specific for c-Myc-deficient mammary glands. These results suggest that there is a general reduction in translation efficiency in mammary glands in the absence of c-Myc.

In addition to Pol II targets, c-Myc controls Pol I-mediated rRNA and Pol III-mediated tRNA and 5S rRNA transcription, thereby regulating cellular physiology at multiple levels [1,47-49]. Accordingly, we analyzed a panel of Pol I, II and III c-Myc targets implicated in ribosome biogenesis and translation. The results from qPCR are displayed as relative expression levels in mutant mice, compared with matched WT littermates; the data are from two pairs of mice at the indicated times in lactation (Table 1). mRNAs encoding nucleolin and nucleophosmin, which are involved in ribosome biogenesis, mRNAs encoding large and small ribosomal subunit proteins, and the mRNA for poly(A)-binding protein1 (PABPC1), involved in translation, all showed a decrease in samples from mutant females. In particular, the ribosomal protein encoding mRNAs were strongly affected, frequently being more than two-fold downregulated in c-Myc-deficient glands (Table 1, values below 0.50). Furthermore, the levels of 5S rRNA as well as the rapidly processed 5'-external transcribed spacer of the 45S rRNA precursor [7], were generally lower in c-Myc mutant glands. This suggests that the decreased translation efficiency in c-Myc mutant glands is due to a general impairment of ribosome biogenesis and translation.

**Table 1:** Levels of c-Myc targets involved in ribosome biogenesis and translation

**Targets**	**Relative expression^a^**			
	
	**L2.5**		**L5.5**	
		
	**Mu-1**	**Mu-2**	**Mu-3**	**Mu-4**
**RNA Pol II products^b^**				
Nucleolar proteins				
Nucleophosmin	0.62	0.78	0.59	0.39
Nucleolin	0.58	0.93	0.65	0.26
				
Large ribosomal proteins				
L3	0.47	0.77	0.40	0.24
L6	0.56	1.08	0.44	0.32
L11	0.62	0.97	0.37	0.33
L23	0.73	0.59	0.36	0.25
				
Small ribosomal proteins				
S3	0.46	0.55	0.60	0.22
S19	0.49	0.83	0.45	0.18
				
Other				
Poly(A)-binding protein	0.60	0.73	0.74	0.56
				
**RNA Pol I product**				
5'-external transcribed spacer of 45S pre-rRNA	0.53	1.19	0.29	0.79
				
**RNA Pol III product**				
5S rRNA	3.08	0.41	0.28	0.38

^a^Results are relative expression levels in mutant mice compared with levels in the corresponding wild type (WT) littermates. Four mutant mice (Mu-1 to -4) were analyzed on lactation day 2.5 and 5.5 and each value is relative to the matched WT value. ^b^Direct targets of transcription activated by c-Myc, chosen from [46].

Finally, we examined the translational efficiency, that is, ribosomal load, of specific mRNAs using RNA isolated from each fraction of the polysome gradient. The mRNAs encoding Lalba, Csn2, Fads2, Scd2, Elovl1 and Aldo3 each shifted to smaller polysomes, with the peaks in fractions 7 to 9 in mutant versus 8 to 10 in WT glands (Figure 4(c), upper panel, open arrow heads). Interestingly, while each of these transcripts is expressed to the same level in WT and mutant mammary glands (Figure 4(a)), this shift clearly shows that they are less efficiently translated. In contrast to the mRNAs encoding proteins directly involved in milk production, the mRNA distribution of β-actin, CK18 and GAPDH along the polysome gradients was essentially the same in WT and mutant glands (Figure 4(c), lower panel, open arrow heads). To confirm that the observed reduced translation efficiency results in less protein production in mutant glands, we performed a Western analysis for β-casein on mammary gland lysates (Figure 4(d)). Compared with the α-tubulin loading control, there is a clear reduction in casein levels in lysates of mutants compared with WT littermates. Taken together, these results show that a reduction in translation efficiency is likely to be responsible for slower milk production in c-Myc mutant glands.

### Delayed proliferative response in c-Myc mutant mammary glands

**Figure 5:**
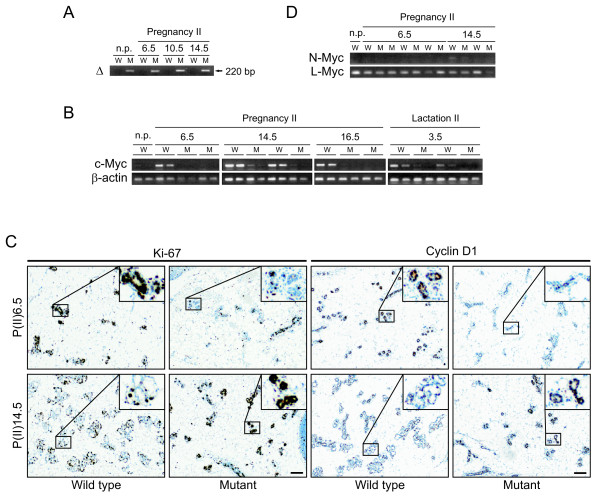
**Delayed proliferative response of c-Myc mutant cells in second pregnancy**. **(a) **Detection of recombined allele in mutant glands as described in Figure 1(c). np = non-pregnant, parous. **(b) **Semi-quantitative reverse transcription-polymerase chain reaction (RT-PCR) on *c-myc *and *β-actin *as described in Figure 1(d). **(c) **Immunohistochemistry for Ki-67 (left) and cyclin D1 (right) on wild type (WT) and mutant glands taken at day 6.5 and 14.5 of a second pregnancy. Ki-67 stains all actively cycling cells (brown), resting cells in G0 are counterstained (blue). Scale bar, 100 μm. **(d) **Expression levels of *N*- and *L-myc *determined via semi-quantitative RT-PCR.

c-Myc loss has an effect on cell cycle progression and proliferation in many organs [25,28,29,31,33]. Thus, we investigated if c-Myc loss affects proliferation during pregnancy. The WAPiCre model is particularly suited for studying proliferation in a second pregnancy since a population of *WAPiCre *expressing cells does not undergo a secretory fate, but survives lactation and involution. These cells are termed Pi-MECs (for parity-identified mammary epithelial cells) (see also Smith and Medina [50]) and function as progenitor cells for epithelium-forming alveolar structures during ensuing rounds of pregnancy and lactation [51,52]. In our model, cells in *c-myc*^*fl*/*fl*^;*WAPiCre*^+ ^mice that survive involution will have lost c-Myc due to Cre expression during the first pregnancy. Consistent with these characteristics, the recombined *c-myc *allele was detected in non-pregnant, parous females, and in all stages of a second pregnancy (Figure 5(a)), in contrast to the first pregnancy where recombination was first detectable at day 14.5 (Figure 1(c)). Furthermore, *c-myc *mRNA levels are very low during a second pregnancy and lactation in mutant, compared with WT glands (Figure 5(b)).

In the normal mammary gland *c-myc *mRNA is highest between day 6.5 and day 12.5 of pregnancy then drops to baseline for the remainder of pregnancy and throughout lactation [53]. In our model, during the first pregnancy Cre activity, hence c-Myc deletion is maximal early in lactation, a time when it has not been possible to detect c-Myc by IHC (data not shown; Klinakis *et al*. [36]). However, since the recombined *c-myc *allele was detected in all stages of a second pregnancy (Figure 5(a)) and *c-myc *mRNA levels are very low in mutant glands (Figure 5(b)), we performed IHC staining for c-Myc on sections prepared from second pregnancy day 6.5 mammary glands. c-Myc staining was evident in sections prepared from WT females (see Additional file 1), although not as strong as the day 10.5 embryonic liver positive control [22]. In contrast, in the mutant glands, c-Myc staining was absent in most of the epithelial clusters. These results clearly show that in *c-myc*^*fl*/*fl*^;*WAPiCre*^+^mice c-Myc mRNA and protein are lost.

To monitor proliferation during pregnancy, IHC for Ki-67, which stains all but G0 cells, was performed (Figure 5(c), left). Furthermore, cyclin D1, which is preferentially expressed in the mammary gland and is essential for proliferation [54] was analyzed by IHC (Figure 5(c), right). In sections from WT glands the majority of cells were actively cycling at pregnancy day 6.5, displaying positive Ki-67 staining, as well as high levels of cyclin D1. In striking contrast, in mutant glands analyzed on the same day, the majority of cells were Ki-67 negative, and had low or undetectable cyclin D1, showing that most cells were not proliferating. By pregnancy day 14.5, however, the majority of mutant cells were cycling, showing that the slower proliferative response was surmountable. Mutant glands at day 14.5 resembled WT glands at day 6.5, whereas by day 14.5, WT glands displayed advanced development with many lumen-forming, alveolar clusters. Of note, levels of *N-myc *and *L-myc *were the same as in WT glands, showing that there was no compensation at the mRNA level in glands lacking c-Myc (Figure 5(d)). In summary, the results indicate that in the absence of c-Myc, alveolar cells show a delayed proliferative response at the start of pregnancy.

### Delayed, but successful differentiation in c-Myc mutant glands

**Figure 6:**
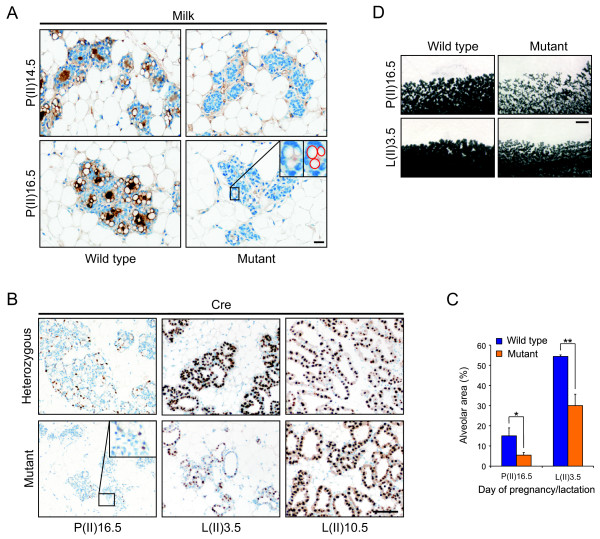
**Delayed differentiation of c-Myc mutant glands in a second pregnancy**. **(a) **Immunohistochemistry (IHC) against mouse milk in glands taken from wild type (WT) and mutant mice at second pregnancy day 14.5 and 16.5. Milk proteins are stained in brown, lipid droplets in the magnification are circled in red. Scale bar, 20 μm. **(b) **IHC against Cre on heterozygous and mutant glands taken at the indicated time points of a second pregnancy and lactation as described in Figure 1(b). Scale bar, 100 μm. **(c) **Quantification of alveolar area in glands as described in Figure 2(c). Three and two pairs of animals (two sections per animal) were quantified at second pregnancy day 16.5 and lactation day 3.5, respectively. *, *P *= 0.0082; **, *P *= 0.013. **(d) **Whole mounts of WT and mutant mice at second pregnancy day 16.5 and lactation day 3.5. Scale bar, 1 mm.

The slower proliferation in the mutant glands resulted in delayed differentiation, which was monitored by IHC against milk proteins (Figure 6(a)). While the WT gland at pregnancy day 14.5 was producing milk, as shown by the milk-filled lumen of the alveoli, the small alveolar clusters in the mutant gland were essentially empty and no milk was detected. By pregnancy day 16.5, however, alveoli occasionally contained cytoplasmic lipid droplets (Figure 6(a), red circles, insert), indicating that the mutant cells had begun differentiation and milk production. Indeed, despite the delay in development, milk production was successful since mutant mothers were able to nurse their pups after parturition, albeit with pups showing reduced body weight (data not shown). Interestingly, expression of the *WAPiCre *transgene was also delayed in a second pregnancy (Figure 6(b)). A control female (*c-Myc*^*fl*/+^;*WAPiCre*^+^) in its second pregnancy and lactation showed scattered Cre staining at pregnancy day 16.5 and ubiquitous staining at lactation day 3.5 (as in Figure 1(b)). In contrast, mutant glands displayed almost no Cre-expressing cells at day 16.5 of a second pregnancy and only scattered positive cells at lactation day 3.5. Importantly, at day 10.5 of the second lactation, the mutant glands showed ubiquitous Cre expression, indicating that the transgene had not been silenced (Figure 6(b)). These results suggest that in the c-Myc mutant glands the *WAPiCre *transgene and endogenous milk protein genes show a similar delay in their expression pattern, very likely reflecting the slower proliferative response.

Finally, quantification of the alveolar density showed that during a second round of pregnancy and lactation, c-Myc mutant glands displayed a strongly reduced alveolar area (Figure 6(c)). With more than a 40% reduction on lactation day 3.5, this effect is more severe than the 30% decrease observed in a first pregnancy (Figure 3(b)). The reduced alveolar area is also evident in whole mount preparations from WT and mutant females obtained at the same time points (Figure 6(d)). The results might be explained, in part, by the slower proliferation leading to an incomplete alveolar expansion in the mutant glands (Figure 5(c)). In conclusion, the data suggest that c-Myc is dispensable for secretory differentiation, however, due to slower proliferation there is also a delay in differentiation in c-Myc mutant mammary glands.

### Effects on progenitor cells in c-Myc mutant glands

**Figure 7:**
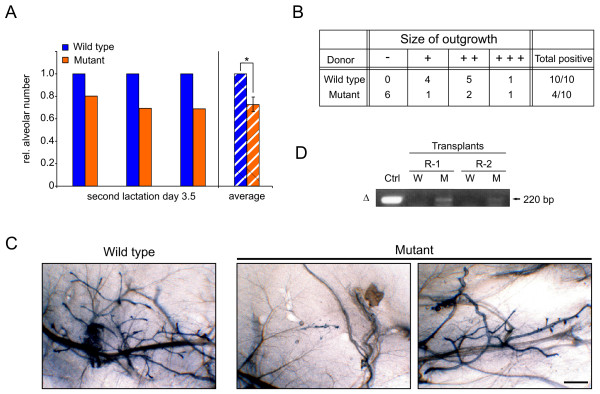
**Effects on mammary progenitor cells**. **(a) **Number of alveoli (including mean ± standard deviation) measured in three pairs of animals at second lactation day 3.5, which were used for area measurement in Figure 6(c). Numbers were calculated per analyzed area, and values from wild type (WT) glands were set as 1. *, *P *= 0.018. **(b) **Summary of two transplantation experiments with a total of 10 NOD/SCID mice grafted contralaterally with WT and mutant epithelium. Positive outgrowths were classified as '+' (filling <25% of cleared gland), '+ +' (filling 25% to 50%) or '+ + +' (filling about 75%). **(c) **Wild type: whole mount of virgin recipient gland containing a '+ +' outgrowth from transplanted WT epithelium, 8 weeks after transplantation. Mutants: whole mounts of a negative and a positive ('+ +') outgrowth obtained from mutant epithelium (left and right panel, respectively). Scale bar, 1 mm. **(d) **Polymerase chain reaction for the recombined allele as described in Figures 1(c) and 5(a), performed on DNA isolated from two recipient mice bearing WT (W) and positive ('+ +') mutant (M) outgrowths. Control: lactating mutant control; R-1, R-2: NOD/SCID recipient 1 and 2.

Considering the more severe phenotype in the second pregnancy, we performed additional experiments to investigate the role of c-Myc in mammary progenitor cells. A quantification of the alveolar number from three pairs of WT and mutant females, analyzed at lactation day 3.5 of a second pregnancy, revealed a significant reduction in c-Myc mutant glands (Figure 7(a)), suggesting that mutant glands start the second pregnancy with fewer alveolar progenitor cells. Importantly, there was no difference in the alveolar number between WT and mutant glands measured at the first lactation (Figure 3(c)).

To functionally investigate mammary progenitor cells, we performed reconstitution experiments into cleared mammary fat pads. Pieces of mammary glands from WT and mutant mothers were transplanted into NOD/SCID recipients. Donor glands were taken from lactation day 5.5, a time point when Cre activity is maximal and most cells will have lost c-Myc. Recipients were sacrificed after 8 weeks in order to examine survival and outgrowth potential of mammary progenitor cells. The results from two independent experiments are summarized in Figure 7(b). Epithelium from WT donors reconstituted a ductal network in all recipients. A representative outgrowth that filled around 30% of the gland ('+ +') is shown in Figure 7(c). In contrast, in 60% of the cases, transplanted epithelium from mutant donors failed to grow out and only rudimentary ductal trees were detected in the recipients (Figure 7(c), mutant, left panel). In the cases when mutant epithelium formed ductal outgrowths (Figure 7(c), mutant, right panel), these were similar to those formed by WT epithelium. A PCR analysis showed that the recombined allele could be detected in DNA recovered from two positive ('+ +') mutant outgrowths (Figure 7(d)), showing that c-Myc-deficient epithelial cells survived and likely contributed to outgrowth formation. In conclusion this suggests that c-Myc has an impact on mammary gland progenitor cell survival and/or proliferation.

## Discussion

Since the early 1980s, numerous investigations focused on c-Myc, exploring its role in normal organ physiology, as well as in tumor biology (for recent reviews see Eilers and Eisenman [1], Meyer and Penn [55]). Results from mammary gland transgenic models implicate c-Myc with lineage commitment during embryonic development [56], with precocious proliferation and differentiation during pregnancy [57], and with premature involution [58]. c-Myc has also been intensely studied in breast cancer [16,59,60], and in mouse models of mammary cancer [17-19]. Here we present for the first time physiological functions of c-Myc during mammary gland development using a conditional knockout approach. Given the ability of c-Myc to regulate transcription of a large number of genes, thereby impacting on all aspects of cellular physiology, it is not surprising that loss of c-Myc in the mammary gland affects different processes. We observed strong phenotypes at the start of pregnancy and during lactation; whereas during involution no alterations in the c-Myc mutant glands were observed (data not shown). At the start of pregnancy, c-Myc-deficient alveolar cells were impeded in their proliferative response, resulting in a delayed ability to differentiate. Moreover, mutant glands displayed slower milk production, a general decrease in translation and reduced expression levels of ribosomal RNA and proteins. Finally, the mutant mammary epithelium had a reduced ability to grow when transplanted into mammary fat pads suggesting that c-Myc has a role in progenitor cell proliferation and/or survival.

### Role of c-Myc in proliferation at pregnancy

Pregnancy is a time of intense cell division, and c-Myc levels increase early in this developmental phase (our observations; Master *et al*. [53]). Indeed, cells from WT females are essentially all cycling, showing high levels of cyclin D1 early in pregnancy. In contrast, c-Myc-deficient alveolar cells remained in G0, displaying lower levels of cyclin D1, and were delayed by at least 6 days in their proliferation. The extensive alveolar development occurring during the first half of pregnancy is dominated by progesterone (for reviews see Naidu *et al*. [61], Neville *et al*. [62]) and c-Myc might have a role in mediating the response to this steroid hormone. In breast cancer models, progesterone induces c-Myc expression [63] via a progesterone receptor regulatory element upstream of *c-myc *[64]. c-Myc has a well-described role in cell growth and proliferation [1]. Thus, one mechanism underlying the slower proliferation might be related to the established role of c-Myc in controlling expression of cell cycle regulators [65], and progesterone might be the upstream regulator of c-Myc. Moreover, c-Myc effects on proliferation might be more indirect by regulating production of paracrine factors, many of which have been shown to be required during alveolar development (see, for example, Naidu *et al*. [61]). It should also be mentioned that since this phenotype was observed in the second pregnancy, it might result from a secondary effect of reduced translation and biosynthetic activity during preceding developmental stages. While c-Myc has been described to couple cell growth to cell division [66], the question whether the observed effect in the mammary gland is secondary or intrinsic to c-Myc loss, can be better addressed with alternative Cre models.

During the second half of pregnancy, c-Myc-deficient cells were proliferating and the mutant gland did 'catch up' with the WT, as attested to by the ability of mutant mothers to nurse. That this alveolar development is due to c-Myc-proficient 'escaper' cells is very unlikely, since at pregnancy day 14.5 c-Myc levels are still very low while Cre expression and recombination only re-starts at day 16.5. An obvious reason explaining this phenotype might be a slow compensation for c-Myc loss by other Myc family members, since they are, in part, functionally redundant to c-Myc [22,33]. While this cannot be ruled out, there was no observable increase in *L-myc *or *N-myc *expression at day 14.5 of pregnancy, a time point when mutant cells were dividing. While we can only speculate, it is possible that this developmental stage proceeds independently of c-Myc. Indeed, the second half of pregnancy is controlled by ligands activating prolactin receptor signaling [62], and the Elf5 transcription factor was shown to be a key mediator of prolactin receptor signaling in promoting alveolar development [67]. Thus, we propose a model whereby c-Myc is required early in pregnancy, potentially downstream of progesterone signaling, but is dispensable for alveologenesis during the second half of pregnancy.

### Role of c-Myc in translation during lactation

We also studied the role of c-Myc during lactation, when the gland devotes its energy to the coordinately regulated process of milk production. mRNAs encoding various milk proteins and enzymes that are strongly upregulated during lactation [43,44] were found at similar levels in WT and mutant mammary glands, suggesting that loss of c-Myc does not impair their transcription. Furthermore, milk produced by the mutant glands is identical in composition to that made in control glands and pups were healthy, albeit with a slower weight-gain when nursing on mutant mothers. This phenotype, suggesting that there was a slower rate of milk production in the mutant glands was investigated and shown to result from a general decrease in translation efficiency.

Numerous studies have described c-Myc's multifaceted roles in mRNA translation, either via transcriptional [48] or non-transcriptional mechanisms [13]. Our results show that c-Myc controls transcription of various target genes with important roles in translation. The c-Myc mutant glands displayed lower levels of mRNA encoding PABPC1, which is involved in translation initiation, and mRNAs encoding nucleophosmin and nucleolin, both involved in ribosome biogenesis. Interestingly, c-Myc loss in intestinal crypts also led to reduced biosynthetic activity, characterized by a loss of nucleolar organizing regions and decreased expression of nucleophosmin [31]. Moreover, very compelling results showing c-Myc's importance during lactation arose from our examination of ribosomal RNAs, and mRNAs encoding ribosomal proteins; these showed a marked reduction in c-Myc mutant glands. Thus, c-Myc is needed for efficient Pol I, II and III transcription in the mammary gland. Since ribosome availability is the rate-limiting step in protein synthesis [68], the strong decrease in the RNA level of components needed for ribosome biogenesis, combined with others important for translation, very likely explains the reduced milk production in the c-Myc mutant glands. Although we did not examine cell size in the mammary gland, it is possible that the reduced biosynthetic activity could also result in smaller cells, as c-Myc regulates cell size in some models/organs analyzed [25,26,31,69]. It will be interesting to address this aspect in detail in future studies.

Hormonal induction of milk production during lactation is subject to transcriptional and translational control mechanisms (reviewed in Rhoads and Grudzien-Nogalska [70], Rosen *et al*. [71]); the latter have been extensively studied using polysome fractionation techniques [72,73]. Here we show that c-Myc has a general role in translation efficiency, as attested to by the reduction in the average size of the polysomes in its absence; however, some selectivity was also uncovered. While mRNA transcripts of milk proteins (Lalba and Csn2) and enzymes important for milk production (Fads2, Scd2, Elovl1, and Aldo3) were shifted to smaller polysomes in c-Myc mutant glands, other mRNAs were not affected. Indeed, there was little or no change in the ribosome loading of *β-actin*, *CK18*, and *GAPDH *mRNAs in the absence of c-Myc, suggesting that their translation efficiency is not altered. It is well established that different categories of mRNAs, referred to as 'weak' and 'strong', have diverse responses to general changes in translation [74] and mRNAs for house-keeping proteins are the least affected by external stimuli. Thus, one plausible mechanism underlying the translational selectivity could be that during lactation, a time when the energy of the organ is devoted to milk production, targets upregulated during the differentiation program would be most affected by the limited availability of ribosomes, while other mRNAs continue to be translated with constant ribosome occupation. Finally, a recent study showed that c-Myc stimulates translation by enhancing mRNA cap methylation and subsequent ribosomal loading [13]. This mechanism might also contribute to the selectivity that we uncovered in the mammary gland. In conclusion, we show here that c-Myc-deficient glands have reduced levels of ribosomal proteins and RNA, as well as proteins involved in ribosome biogenesis and translation. Therefore, by acting on many different components of the translation machinery, c-Myc has an important role in successful and efficient translation during lactation.

### Role of c-Myc in progenitor cell proliferation/survival

The role of c-Myc in stem and progenitor cells has been intensively studied in different mouse models. In the bone marrow, two reports showed that loss of c-Myc leads to an accumulation of hematopoietic stem cells and a severe loss of the committed lineages due to impaired differentiation [32,34]. Moreover, elimination of both c-Myc and N-Myc in hematopoietic stem cells impairs self-renewal and leads to rapid apoptosis of stem cells [33]. In the skin, depletion of the epidermal stem cell population, due to insufficient amplification of the cells was observed following c-Myc deletion [25]. A function for c-Myc in mammary stem or progenitor cells seems likely, as the Wnt and Notch signaling pathways are believed to play important roles in mammary stem cells [75], and both can directly stimulate c-Myc expression [76].

Indeed, our results suggest that c-Myc plays a role in a subset of mammary gland progenitor cells, the Pi-MECs. This population of WAPiCre expressing cells does not undergo a secretory fate, but survives lactation and involution [51,52]. A model of the mammary stem cell hierarchy suggests that the Pi-MECs are contained in the Sca-1 negative population and function as alveolar progenitors during pregnancy [77]. As c-Myc mutant glands show a reduced number of alveoli in the second pregnancy/lactation, it is likely that fewer progenitor cells were present following c-Myc loss in the first pregnancy and lactation. This hypothesis is further supported by results from the transplantation experiments. The reduced outgrowth capacity found with the mutant epithelium suggests that alveolar progenitors have an impaired ability to proliferate or to survive in the absence of c-Myc.

### Relevance of c-Myc in cancer

The importance of c-Myc in cancer was established more than 20 years ago and much effort has gone into studying all aspects of oncogenic c-Myc (reviewed in Meyer and Penn [55]). c-Myc is aberrantly expressed in most breast cancers as a result of gene amplification or from alterations in signaling pathways that impact on c-Myc RNA or protein levels. Myc and many of its target genes were recently shown to be strongly expressed in basal, ERα negative breast tumors, allowing them to proliferate in the absence of estradiol-induced signaling [78]. Our studies on c-Myc in normal development have important implications for breast cancer. Indeed, it was shown in a c-Myc-induced tumor model that Myc's ability to increase protein synthesis was a major factor contributing to aberrant growth and genomic lesions [79].

Despite the phenotypes in pregnancy and lactation, the effects of c-Myc loss in the mammary gland are generally well tolerated, which is of interest considering c-Myc as a target in cancer therapy [14,80]. Recent studies have evaluated the role of c-Myc in tumor onset and maintenance and have also addressed side effects of Myc-targeting. In the intestines, where inactivation of adenomatous polyposis coli (APC) is a key event in colorectal cancer development, c-Myc is frequently overexpressed as a downstream β-catenin/T cell factor target. Interfering with c-Myc levels in mouse models with APC mutations rescued the observed phenotypes, leading to a reduction in tumor burden and increased survival [81,82]. Furthermore, by employing an inducible, dimerization-interfering Myc construct in a Ras-induced lung adenocarcinoma model, it was shown that Myc inhibition impaired tumor maintenance. Importantly, the 'side-effects' observed in other organs disappeared rapidly after cessation of Myc inhibition [83]. Finally in Notch1-induced mammary tumors it was shown that ablation of c-Myc reduces tumor incidence and increases tumor latency, suggesting that Myc might be an attractive target in cancers with deregulated Notch signaling [36]. Multiple signal transduction pathways activate c-Myc [84], many of which are deregulated in breast cancer [85-87]. Future studies using different mammary tumor models will provide more insight into the role of c-Myc in tumor development and maintenance, and in its potential as a breast cancer target.

## Conclusion

Our data revealed three interesting new roles for c-Myc in the mouse mammary gland. At the start of pregnancy, c-Myc loss resulted in delayed proliferative response and differentiation. During lactation, mutant glands showed reduced milk production and slower pup weight-gain. Furthermore, c-Myc-deficient glands were generally impaired in translation efficiency and displayed reduced levels of ribosomal RNA and proteins. Finally, the results from transplantation assays suggest that c-Myc has a role in progenitor cell proliferation and/or survival. Our results provide new insight into Myc's physiological role in breast development, which might gain special importance considering c-Myc as a novel target in the aggressive basal breast cancer subtype.

## Methods

### Mouse strains

*c-myc*^*fl*/*fl *^mice were mated with mice containing a single copy of the WAPiCre transgene and pups were further intercrossed. Littermates with the genotype *c-myc*^*fl*/*fl*^;*WAPiCre*^- ^or *c-myc*^*fl*/+^;*WAPiCre*^- ^(referred to as WT), *c-myc*^*fl*/*fl*^;*WAPiCre*^+ ^(mutant) and *c-myc*^*fl*/+^;*WAPiCre*^+ ^(heterozygous) were used for all studies. Mothers were maintained with litters of six pups and only inguinal glands were taken in the experiments. For growth analysis, newborn pups were mixed and two to seven pups were placed with WT and mutant mothers. Body weight of each pup was measured regularly and the results presented as average weight ± standard deviation. For milk volume experiments, mothers were either directly sacrificed or after a 2 hour period without pups, to allow milk filling of the gland. For milking females, pups were removed from mothers for at least 4 hours, then mice were anaesthetized with Ketarom (100 μl/10 g body weight intraperitoneal) and milk release was induced by intraperitoneal injection of 0.3 IU oxytocin. Milk was removed by applying gentle pressure and directly drawing it into capillary tubes or pipettes for further analysis. All animal experiments were carried out under the Swiss guidelines for animal safety.

### DNA/RNA isolation, PCR and RT-PCR

Pieces of mammary glands were flash frozen in liquid nitrogen for RNA or DNA isolation. DNA was precipitated with ethanol after proteinase K digestion (56°C overnight). For detection of the recombined *c-myc *allele, the following primers were used in a PCR: fw: 5'-AAATAGTGATCGTAG-TAAAATTTAGCCTG-3'; rw: 5'-TACAGTCCC-AAAGCCCCAGCCAAG-3'. RNA was prepared using TRIzol reagent (Invitrogen, Carlsbad, CA, USA) according to the manufacturer's instructions. Reverse transcription was carried out using the Ready-To-Go You-Prime First-Strand Beads (GE Healthcare, Buckinghamshire, UK) with oligo(dT) 15 or random hexamer primers (both Promega, Madison, WI, USA) for mRNA or rRNA detection, respectively. We used 2 μl and/or 4 μl cDNA for semi-quantitative PCR analysis. Detailed information (including primers for semi- and quantitative PCR) can be found in Additional file 2.

### Milk analysis

Aliquots of milk were centrifuged in capillary tubes (30 minutes, 3,500 rpm) to determine the fat content (creamatocrit), measured as the ratio of the upper cream layer length over total milk length [42]. Milk protein composition was analyzed by diluting fresh milk 1:20 in phosphate-buffered saline (PBS) and loading 5 μl and 10 μl on 15% SDS-PAGE, which was stained with Coomassie Blue. For measuring lactose content, samples of milk, frozen in liquid nitrogen and stored at -80°C, were thawed and centrifuged (20 minutes, 4°C, 3,000 g) and 10 μl of the lower aqueous phase were used in a colorimetric galactose/lactose assay-kit (BioVision, Mountain View, CA, USA).

### Immunohistochemistry

For histological examination, the central region of inguinal mammary glands containing the lymph node was used. Glands were fixed in freshly prepared 4% paraformaldehyde in PBS and stored in 70% ethanol until embedding in paraffin. IHC was performed on 4 μm paraffin sections using the following antibodies: Cre [35], CK18 (Progen Biotechnik, Heidelberg, Germany), c-Myc (Upstate Biotechnology, 06-340, Lake Placid, NY, USA), Ki-67 (Lab Vision, Fremont, CA, USA), cyclin D1 (Cell Marque, Rocklin, CA, USA) and rabbit anti-milk serum [41]. Stainings were carried out with the Discovery XT Staining Module (Ventana Medical Systems SA, Strasbourg, France). Images were acquired with a Leica DFC420 camera on a Nikon Eclipse E600 microscope using Plan Fluor 10×/0.3, 20×/0.5, and 40×/0.75 lenses. When necessary, optimization of brightness and contrast was performed by standard procedures in Corel DRAW 13 and always applied equally to the whole set of images.

### Mammary gland whole mounts and electron microscopy

For whole mount staining, inguinal glands were spread on glass slides, fixed overnight at 4°C in Tellyesniczky's fixative and stained with iron-hematoxylin as described . Images were captured by a Leica DFC420 camera on a Nikon Eclipse E600 microscope with a Plan Apo 4×/0.2 lens in the milk-filling experiments. Pictures of other whole mounts and transplants were taken on a Leica Z6 APO A microscope with a Plan Apo 2.0× lens and a Leica DFC480 camera.

For electron microscopy, pieces of mammary gland were fixed in Karnovsky's fixative (3% paraformaldehyde, 0.5% glutaraldehyde in 10 mM PBS pH 7.4), washed, and post-fixed in 1% OsO_4_. After dehydration with graded series of ethanol, samples were embedded in Epon and sections of 60 to 70 nm thickness were cut. Sections were double stained with uranyl acetate and lead acetate [88] and viewed in a FEI Morgagni 268D transmission electron microscope.

### Image analysis and statistics

Images were taken with a Mirax Slidescanner (Zeiss AG, Zurich, Switzerland) using a 20×/0.5 lens (0.2 μm/pixel) and converted into standard TIFF format. Manual counting of alveoli and measurement of alveolar areas were performed on TIFF-files using the measurement module of ImageAccess (Imagic AG, Glattbrugg, Switzerland). For automatic detection and measurement of alveolar area versus total organ area, images taken with the same slidescanner were analyzed using Definiens Software (Definiens AG, Munich, Germany).

Statistical analysis for alveolar area quantification and pup weight analysis was performed with one-sided Student's *t*-test. For alveolar counts, the ratios of the numbers obtained from mutant versus corresponding WT littermate were tested for significant deviation from one using 'one sample' *t*-test.

### Lysate preparation and Western blot analysis

Frozen pieces of mammary gland were ground to powder in liquid nitrogen and homogenized in RIPA buffer (50 mM Tris pH8, 1% NP40, 0.5% sodium deoxycholate, 20% SDS, 150 mM NaCl) complemented with 5 mM ethylene glycol tetraacetic acid, 1 mM dithiothreitol (DTT), 20 mM sodium pyrophosphate, 10 μg/ml aprotinin, 10 μg/ml leupeptin, 0.5 mM phenylmethanesulfonylfluoride (PMSF) and 1 mM sodium orthovanadate. Lysates were subjected to SDS-PAGE and transferred to polyvinylidene fluoride membranes (Millipore, Bedford, MA, USA). After blocking, membranes were incubated overnight at 4°C with primary antibodies against α-tubulin (Neomarkers, Fremont, CA, USA) and β-casein [89], or 1 hour at RT with rabbit anti-milk serum [41]. Signals were detected by using horseradish peroxidase-linked secondary antibodies (GE Healthcare) and enhanced chemiluminescent detection reagent (GE Healthcare).

### Polysome fractionation of mammary glands and livers

Half of an inguinal mammary gland and a piece of liver (both approximately 80 to 120 mg) were flash frozen in liquid nitrogen. For extract preparation, tissue was ground to a white homogeneous powder in liquid nitrogen with 1 ml polysome buffer (10 mM Tris pH8, 150 mM NaCl, 5 mM MgCl_2_, 1% NP-40, 1% DOC, 10 mM DTT, 50 μg/ml cycloheximide, 0.4 U/μl RNAsin, 1 mM PMSF, 20 μg/ml aprotinin and leupeptin, supplemented with complete protease inhibitor (Roche Diagnostics, Indianapolis, IN, USA)). After thawing, cell debris were removed by centrifugation (12,000 g, 10 minutes, 4°C) and 700 μl of supernatant were loaded on to a linear sucrose gradient (15% to 60% sucrose (w/v), in 10 mM Tris pH 7.5, 140 mM NaCl, 1.5 mM MgCl_2_, 10 mM DTT, 100 μg/ml cycloheximide). Gradients were centrifuged in a SW41Ti rotor (Beckman Coulter Inc., Fullerton, CA, USA) for 2 hours at 38,000 rpm at 4°C with brakes off. Twelve fractions of 0.5 ml were collected as previously described [90]. RNA was isolated using TriZol reagent as described above: 1 μl glycogen (20 mg/ml) was added to facilitate isopropanol precipitation of RNA.

### Transplantation of mammary epithelium into cleared fat pads of NOD/SCID recipients

Inguinal mammary glands of 3- to 4-week-old NOD/SCID mice (body weight below 13 g) were cleared of endogenous mammary epithelium as described [91]. Donor epithelium was derived from mammary glands of day 5.5 lactating WT and mutant mothers and chopped into approximately 1 mm^3 ^pieces. Outgrowth efficiency was monitored 8 weeks after transplantation by sacrificing non-pregnant recipients and staining mammary gland whole mounts as described above. Transplants were scored as successful when originating from a central part of the cleared gland with ducts growing in all directions [92]. Positive outgrowths were rated as '+' (filling <25% of the gland), '+ +' (filling 25% to 50%) and '+ + +' (filling about 75%).

## Abbreviations

APC: adenomatous polyposis coli; CK: cytokeratin; DTT: dithiothreitol; IHC: immunohistochemistry; PBS: phosphate-buffered saline; PCR: polymerase chain reaction; PMSF: phenylmethanesulfonylfluoride; RT-PCR: reverse transcription-polymerase chain reaction; WT: wild type.

## Authors' contributions

TS participated in the design of the study, carried out all of the experimental work and analyzed the data. PS designed and developed the image analysis strategy. AT participated in designing the study and provided the *c-myc*^*fl*/*fl *^mice. NEH conceived the study and participated in its design and coordination. TS and NEH wrote the manuscript. All authors read and approved the final manuscript.

## Supplementary Material

Additional file 1 **Additional figure**. Immunohistochemistry for c-Myc. **(a) **Fetal liver of a day 10.5 embryo as positive control [22] showing strong nuclear staining in dark violet (counterstain pink). Scale bar, 50 μm. **(b) **Wild type (WT) and mutant mammary glands at second pregnancy day 6.5, when c-Myc expression is highest. WT epithelium shows clear dark staining compared with mutant glands, shown in the upper panel in violet (with pink counterstain) and in the lower panel in red (no counterstain). Note that in the mutant gland (lower panel) some epithelial clusters retained c-Myc (insert b, red staining) while all other clusters are clearly Myc-deficient (insert a, no staining). Scale bars, 50 μm. Click here for file

Additional file 2 **Supplementary methods**. Condition for quantitative polymerase chain reaction (PCR) and all primers used in semi-quantitative and quantitative PCR analyses. Click here for file
