# Supplementary figures and images for: c-Myc affects mRNA translation, cell proliferation and progenitor cell function in the mammary gland
Source: BMC Biol. 2009 Sep 28;7:63. doi: 10.1186/1741-7007-7-63 (PMC2761394; doi:10.1186/1741-7007-7-63)

Additional Figure: IHC for c-Myc

A

Embryonic liver (day 10.5)

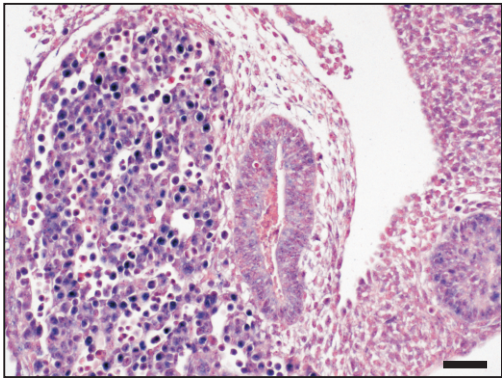

B

Wild type

Mutant

P(II)6.5

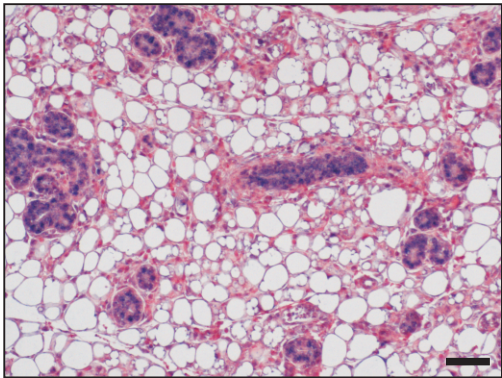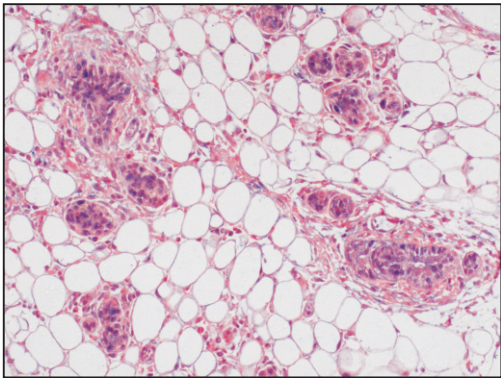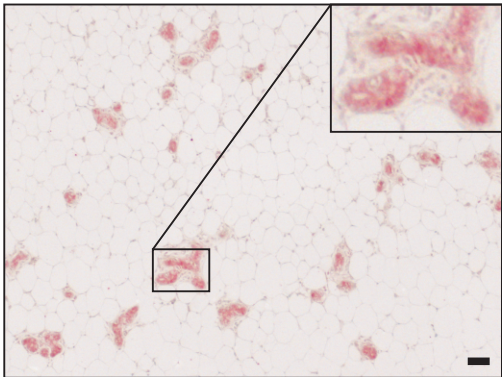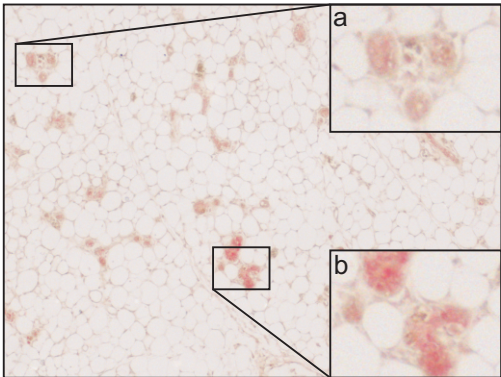

Supplement: Additional file 1 — Additional figure. Immunohistochemistry for c-Myc. (a) Fetal liver of a day 10.5 embryo as positive control [22] showing strong nuclear staining in dark violet (counterstain pink). Scale bar, 50 μm. (b) Wild type (WT) and mutant mammary glands at second pregnancy day 6.5, when c-Myc expression is highest. WT epithelium shows clear dark staining compared with mutant glands, shown in the upper panel in violet (with pink counterstain) and in the lower panel in red (no counterstain). Note that in the mutant gland (lower panel) some epithelial clusters retained c-Myc (insert b, red staining) while all other clusters are clearly Myc-deficient (insert a, no staining). Scale bars, 50 μm. [file 1741-7007-7-63-S1.PDF]
