# Supplementary material for: c-Myc affects mRNA translation, cell proliferation and progenitor cell function in the mammary gland
Source: BMC Biol. 2009 Sep 28;7:63. doi: 10.1186/1741-7007-7-63 (PMC2761394; doi:10.1186/1741-7007-7-63)
Supplement: Additional file 2 — Supplementary methods. Condition for quantitative polymerase chain reaction (PCR) and all primers used in semi-quantitative and quantitative PCR analyses. [file 1741-7007-7-63-S2.PDF]

Stoelzle et al. **Supplementary Methods**

Semi-quantitative and qPCR

Quantitative qPCR was performed using the Absolute SYBR Green ROX Mix (Thermo Scientific, Waltham, MA) and 1 µl of cDNA. Relative expression was determined in a ABI Prism 7000 cycler (Applied Biosystems Inc., Foster city, CA) in duplicate measurements, using *β-actin* levels as reference. All qPCR reactions were performed with the following cycling parameters: 50°C 2 min, 95°C 15 min, 40 cycles of 95°C 15 sec, 60°C 20 sec, 72°C 1 min.

The following specific primers were used for semi-quantitative or quantitative PCR:

Target	Sequence
c-Myc	fw: 5'-GTAATTCCAGCGAGAGACAGAGG-3'
	rw: 5'- TGTTGGTGAAGTTCACGTTGAG-3'
β-actin	fw: 5'-TGCGTGACATCAAAGAGAAG-3'
	rw: 5'-GATGCCACAGGATTCCATA-3'
$p21^{Cip1}$	fw: 5'-GCAAAGTGTGCCGTTGTC-3'
	rw: 5'-AGACCAATCTGCGCTTGG-3'
Lalba	fw: 5'-CCATTAAAGACATAGATGGCTATC-3'
	rw: 5'-CACTGTTCAAGCTTCTCAGAGCAC-3'
Csn2	fw: 5'-ACTACATTTACTGTATCCTCTGAC-3'
	rw: 5'-TGCTACTTGCTGCAGAAAGTACAG-3'
Fads2	fw: 5'- TCCTCTCGTACTTCGGCACT-3'
	rw: 5'- TCTTTATGTCCGGGTCCTTG-3'
Scd2	fw: 5'-ACAACTACCACCACGCCTTC-3'
	rw: 5'-GCTTCTGGAACAGGAACTGC-3'
Elovl1	fw: 5'- CCTAAGTGCCTCAGGACTGC-3'
	rw: 5'- CAGCCCTGAGTGTTCCTCTC-3'
Aldo3	fw: 5'-AACTGGGGCCCTAACTCTGT-3'
	rw: 5'-CCGACAACTCCTTCTTCTGC-3'

CK18	fw: 5'-CAGCTACCTAGACAAGGTGAAG-3'
	rw: 5'-GCCTTGTGATGTTGGTGTCATC-3'
GAPDH	fw: 5'-TTCATTGACCTCAACTACATG-3'
	rw: 5'-GTGGCAGTGATGGCATGGAC-3'
Nucleophosmin	fw: 5'-TCCTGGAGGTGG-TAACAAGG-3'
	rw: 5'-ACCCTTTGATCTCGGTGTTG-3'
Nucleolin	fw: 5'-TTGTACGTGCTCCAGAGTCG-3'
	rw: 5'-TGAGGGATGACAACCTCCTC-3'
RPL3[a]	fw: 5'-GATGACACAGGCAAGAAGCA-3'
	rw: 5'-ATCTCATCCTGCCCAAACAC-3'
RPL6	fw: 5'-AGCGCCTGATACAAAGGAGA-3'
	rw: 5'-TCTCACCAGGACAGGGTTTC-3'
RPL11	fw: 5'-GGGAGTATGAGTTGCGGAAA-3'
	rw: 5'-CCTCCTCCTTGCTGATTCTG-3'
RPL23	fw: 5'-GGCATGACCTTCATGACCTT-3'
	rw: 5'-GTACCTGGTCCCAGCAAAGA-3'
RPS3	fw: 5'-CTGGGACCCAAGTGGTAAGA-3'
	rw: 5'-CCCTCCAGCTTAAACCAACA-3'
RPS19	fw: 5'-TACACACGAGCTGCTTCCAC-3'
	rw: 5'-CGATCCTGTCCAGGTCTCTC-3'
PABPC1	fw: 5'-GTCTCTCCGCTCAAAGGTTG-3'
	rw: 5'-GCTAGACCTGGCATTTGCTC-3'
N-Myc	fw: 5'-GTCGTCGAGTGCTAGCCACAC-3'
	rw: 5'-CTCGTCATCCTCATCATCTGA-3'
L-Myc	fw: 5'-CATGAAGCACTTCCATATCTC-3'
	rw: 5'-GTTCTTCCTCTTGGTCACGTC-3'
5'-ETS[b]	fw: 5'-CTCTTCCCGGTCTTTCTTCC-3'
	rw: 5'-TGATACGGGCAGACACAGAA-3'
5S rRNA	fw: 5'-TGTTTGTGTGGAAGCTGAGG-3'
	rw: 5'-AAAGGAGGACGGCTAGAAGG-3'

[a]RP: ribosomal protein

[b]5'-ETS of the 45S rRNA precursor
